# Homogeneity of *Mycoplasma agalctiae* vaccine strains in an agalaxy- high-burden environment

**Published:** 2019-02

**Authors:** Khatereh Kabiri, Seyyed Ali Pourbakhsh, Jamileh Norouzi, Mohammad Sekhavati, Keyvan Tadayon

**Affiliations:** 1 Department of Microbiology, North Tehran Branch, Islamic Azad University, Tehran, Iran; 2 Agricultural Research, Education and Extension Organization (AREEO), Razi Vaccine and Serum Research Institute, Karaj, Iran

**Keywords:** *Mycoplasma agalactia*, Vaccine, Strain, Homoplasy

## Abstract

**Background and Objectives:**

Interrogation of the genomic relations between Iranian *Mycoplasma agalactiae* vaccine strains of Taliqan, Lorestan and Shiraz.

**Materials and Methods:**

Two MLVA (covering VNTR loci of 5, 9, 17 and 19) and MLST (comprising *dnaA, gltX, gyrB,* C, *tufA* genes) genotyping systems plus nucleotide structure analysis of P80 gene, was conducted.

**Results:**

The shared MLVA pattern represented by the three strains differed to that of the *Mag* PG2 laboratory strain, only at locus VNTR19 where the PG2 genome hold a 3 bp longer stretch. In MLST analysis, at *dnaA, gltX, gyrB, metS* and *tufA* loci, the three strains displayed alleles 1, 21, 2, 2 and 1, respectively. At, *gltX* locus a new allele (21) was detected where a new sequence type (ST33) was identified. Besides, the trio strains hold an identical nucleotide structure in their ma-mp81 gene.

**Conclusion:**

In explanation, lack of efficient disease control measures, has possibly contributed in evolution of a clone or a few clones that gradually overwhelmed the population over the time. Besides, the similarity between the Iranian and the PG2 strains, might be due to homoplasy or farming exercises such as animal importation. Inclusion of further local isolates in next studies will help to assess these assumptions.

## INTRODUCTION

Contagious agalactia (CA) is a highly infectious disease of small ruminants leading to extensive heavy losses to sheep and goat farming across the world. CA has been known for the last two centuries since its first description in Italy (1817) by Metaxa (1). It is reported from all five continents and is included in the List B of dangerous infections recognized by the International Office des Epizooties (OIE). In clinical view, CA behaves as a syndrome mainly affecting joins, mammary glands and eyes (2).

Four mycoplasma species are known to cause CA (3). These are *Mycoplasma agalactiae* (*Mag*), the world-known classic pathogen both in sheep and goats, *Mycoplasma mycoides* subsp. *capri* (Mmc), *Mycoplasma capricolum* subsp. *capricolum* (Mcc) and *Mycoplasma putrefaciens* (Mp) mainly in goats (3, 4).

CA actively infects sheep and goat populations in Mediterranean basin, Balkan region, Middle East, western Asia, and most of Africa (1).

In the Middle-Eastern Iran, Delpy and co-workers reported success in treatment of CA cases using Stovarsol sodique in early 1930s (5) but actually it was 1959 when the first description of CA clinical cases appeared in the literature (6). Since then numerous studies on microbiology, epidemiology, pathogenicity and control (7–10) of CA have been published by Iranian workers. A number of different vaccine preparations using exotic and indigenous strains of *Mag* have been employed to control the disease. While the national vaccination scheme includes annual application of as many as 20 million doses of agalaxy vaccine, Iran still experiences frequent epidemics of CA every year (8–11).

Current epidemiological knowledge of *Mag* has been recently broadened by application of molecular strategies such as variable number tandem repeats (VNTR), Multilocus sequence typing (MLST) and pulsed field gel electrophoresis (PFGE), Random amplified polymorphic DNA (RAPD) techniques (12). The level of genomic diversity of CA seems to be host-dependent as its populations in sheep are understood to be genetically less heterogenic (13) compared to that of the goats (14).

In the recent years VNTR typing systems have been employed in genetic studies of mycoplasmas including *Mycoplasma hyorhinis* (15), *Mycoplasma hyopneumoniae* (16), *Mycoplasma bovis* (17), *Mycoplasma californicum* (18) and *Mycoplasma mycoides* (19). Similarly, MLST genotyping has been used in epidemiological studies of several Mycoplasma species including *M. agalactiae* (20), *Mycoplasma bovis* (20). *Mycoplasma hyopneumoniae* (21), *Mycoplasma hyorhinis* (15) and *Mycoplasma synoviae* (22).

Molecular mass investigations of serum proteins from CA naturally infected sheep resulted in recognition a handful of immunodominant surface membrane proteins expressed in the early phase of infection (23, 24). One of these proteins, named P80 bearing a molecular mass of 80 kDa, is serum detectable in all sheep naturally infected by wild-type *Mag* isolates. The ma-mp81, the gene encoding P80 consists of 2, 166 nucleotides (24).

In 1940s, three local *Mag* isolates collected from Lorestan, Shiraz and Taliqan were selected for preparation of a bacterin vaccine. This liquid saponified biological has been ever manufactured and used in Iran with an average yearly vaccination rate of 20 million doses over the last decade. Anecdotal records show these three strains had differences in their bio-chemical properties at the time of isolation.

This study was intended to genetically characterize the Iranian trio *Mag* vaccine strains of Lorstan, Shiraz and Taliqan with MLST and MLVA methods. This genomic interrogation was further extended with analysis of their ma-mp81 gene compared to strains from rest of the world.

## MATERIALS AND METHODS

### Bacterial culture.

Three *Mag* vaccine strains of Lorestan, Shiraz and Taliqan, preserved frozen in glass vials at −70°C from the RVSRI bacterial archive, were revived through transfer of 5 ml of the thawed material to two Falcon polyethylen tubes containing 45 ml of PPLO broth supplemented with 15% sterile horse serum. Shaking incubation of the culture vessels (37°C) continued for 72 h until expected turbidity attributed to the bacterial growth was achieved. Culture tubes were centrifuged (3,000 g/5 m) and deposition of each tube was transferred to a 1.5 ml O-ring microfuge tube (containing 500 μl TE buffer). Microtubes were submerged in a boiling water bath and heat-inactivated for 15 m when they were centrifuged again. The supernatant liquid, carrying the bacterial genomic material, was preserved in a fresh microtube and directly used for molecular experiments.

### Confirmatory test.

To authenticate identity of the trio strains, they were subjected to the specific PCR amplification based on the *uvr*C gene using primer pair of MAGAUVRC1-L and MAGAUVRC1-R (25).

### PCR amplification.

Analyses of VNTRs was conducted as previously described by McAuliffe et al. (12). Four VNTR loci of 5, 14, 17 and 19 were selected and targeted in the genetic assay.

The MLST analysis was performed based on five housekeeping genes using primer pairs and PCR amplification protocol developed by the McAulif et al. This scheme focuses on partial sequencing of *dnaA, gltX, gyrB, metS* and *tufA* genes.

For ma-mp81 gene structure analysis, four primer pairs were designed using the online software Primer 3 (https://www.ncbi.nlm.nih.gov/tools/primer-blast/) with the default settings and the *Mag* PG2 genome sequence (GenBank assembly accession: GCA_000063605.1, latest). In order to improve accuracy, designing and selection of primers were conducted in a way that flanking primer pairs shared a relatively large amount of target sequence as a total of 5,000 nucleotides were amplified and sequenced in search for the 2,166-bp long ma-mp81 gene (24).

Four VNTR analysis, PCR primer pairs were: VNTR 5, 5F (5′-AAA GAG AAA GGA AGC TGA A- 3′) and 5R (5′-GGA TCA TTA TCG CTT TTT GA-3′); VNTR 14, 14F (5′-TTG AAA TAT CCG CTT AAG AAA-3′) and 14R (5′-AAT TTG CAT TTA ATG GTG CT-3′); VNTR 17, 17F (5′-TTT AGC TTT TGA TTC AAT ACT TTC-3′) and 17R (5′-AAA GAA TTA TGC GAG CAT TT-3′); and VNTR 19, 19F (5′-TTG CTT CTT GTG CTT CTT TT-3′) and 19R (5′- AAG GGG ATC AAC CAG ATA AT-3′).

For MLST analysis primers of PCR were *dnaA*, F (5′-TAA CGT AAC CCC AAA CTC AC-3′) and R (5′- CAT AAT TCA GGC GTC ATC TT-3′); *gltX*, F (5′- GCC TTG CCT ACA AAT CTT AT-3′) and R (5′- ATA GTT GCT TAA GCG CAA AC-3′) *gyrB* F (5′- CAA TAC ACA TCA ACC TTC CA-3′) and R (5′- AGC AGA GTT ACC TTC GAC AA-3′); *metS* F (5′- GCC TGT AAA TTA GCC CTT CT-3′) and R (5′-TTT TAA CCA AAA ATC AAG CTG-3′); *tufA* F (5′- GAA CAT GAT TAC TGG TGC TG-3′) and R (5′- ACG GCC TTT TTC AAA TTC TA-3′).

The PCR primers in ma-mp81 assay were *Mag* P80L F (5′- ATC AAA GGT GCT TGA GAA ATG G -3′) and R (5′- ATA CTG GGG CAT TAG CAG ACA -3′); *Mag* P80M1 F (5′- GAG TAT GCT GAA GGT GAA AAC TCA -3′) and R (5′- ACC CGC TGT TGA ACC TAT ACC A -3′); *Mag* P80M2 F (5′- AAA CTC AGC AGC ACA AAA CTC G -3′) and R (5′- GCG CCT CAA CTT ACA CCA AT -3′); *Mag* P80R F (5′- AAA TAA GGG CAC TGA AAT TGG TAC A-3′) and R (5′- GCT TCT TTA GCT TTA ATT GTG CCT -3′).

PCR Amplifications were performed using an Eppendorf thermocycler in a final volume of 12 μl. Individual PCR reaction consists of 6 μl of ready-to-use PCR master mix containing 2.5 units of Taq DNA polymerase, 200 mM of each of the four dNTPs and 2 mM MgSO4 (Ampliqon®, Denmark), 1 μl of each forward and reverse primers (5 pm/ μl), 2.5 μl of DNA template plus 1.5 μl of double-distilled water to compensate the volume. In PCR-MLST reactions, an extra amount of MgCl_2_ (0.36 μl from a 50 mM solution) was used. The PCR cycling conditions were 45 sec at 95°C, followed by 30 cycles of 45 sec at 95°C, 45 s at 56°C and 1 min at 72°C, this was finished with a final extension step lasting for 10 min at 72°C. Amplicons were kept at 4°C until analysis. Visualization of aliquots was conducted by electrophoresis on 1.5% agarose gel pre-stained with Red Safe® under UV illumination. The molecular weights were estimated using visual comparison of amplicons against a 100-bp Molecular weight standard (26).

All the PCR amplicons from VNTR, MLST and ma-mp81 gene were sequenced using the same PCR primers at the sequencing facility of Macrogen, South Korea.

Sequencing chromatograms were visually checked and edited, if necessary, with Chromas lite (available at http://www.technelysium.com.au/chromaslite.html). AliView and MAFFT suites were consulted to determine the consensus sequence for each locus and also for assembling, trimming, concatenation and aligning the contigs. Tandem Repeat Finder (http://tandem.bu.edu/trf/trf.html) was employed in order to determine the number of repeats in each VNTR product.

VNTR profiles of strains were recorded as character data using allelic patterns. In MLST analysis, the assembled sequences of strains were subjected to the non-redundant *Mycoplasma agalactiae* MLST database (https://pubmlst.org/magalactiae/) to assign allele numbers and sequence types (STs).

To visualize the genetic relationship between *Mag* vaccine strains from Iran and the *Mag* population representing rest of the world in a single image, an analysis of VNTR findings was conducted based on a maximum-parsimony strategy. This was implemented using BioNumerics, version 6.7 where a matrix comprising 9 characters (5 MLST and 6 VNTR loci) was employed to encode the input data.

To position the Iranian vaccine strains in the global picture of MLST profiles framed by the Mag MLST database, a Burst analysis was conducted using eBurst V3 (http://eburst.mlst.net).

## RESULTS

**Fig. 1.**
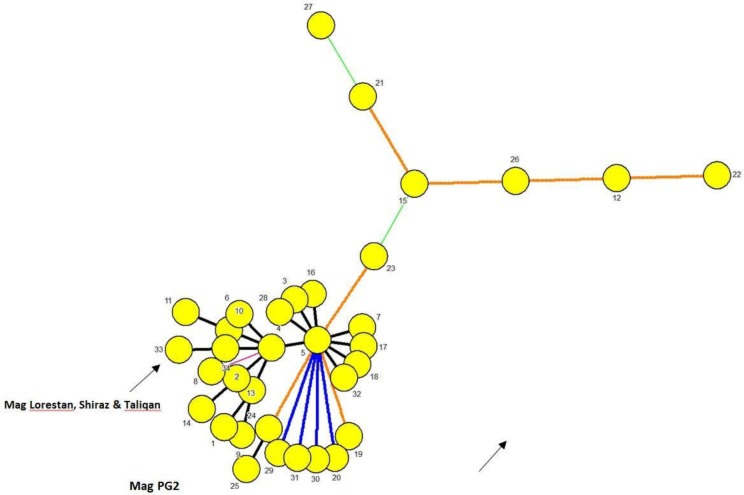
Graphic representation of minimum spanning tree (MST) resulting from MLST typing of the 34 types registered with the international *Mycoplasma agalactiae* MLST Database (https://pubmlst.org/bigsdb?db=pubmlst_magalactiae_seqdef) as of January 2019. An identification policy based on recognition of all taxa with zero inter-taxon distance plus the priority rule using the Maximum number of N-locus variants (N=1) were employed in generation of the MST. Here each MLST type is represented only once. MLST types differing by a single MLST locus are shown by thick short lines while longer thin connecting two MLVA types denote types while those with larger differing loci are denoted by thin longer dashed or dotted lines. The MLST type of the three Iranian and PG2 Ma vaccine and laboratory strains are marked.

In MLST analysis, the three *Mag* strains identically displayed alleles 1, 21, 2, 2 and 1 at d*naA, gltX, gyrB, metS* and *tufA* loci, respectively. All but one of these alleles (the *gltX* allele, 21) were proved to be previously recorded according to the *Mag* MLST database. A new sequence type (ST33) with close similarity to ST4 was assigned to these strains (Fig. 1).

In MLVA analysis, all the three strains identically produced amplicons at VNTR5, VNTR14, VNTR17 and VNTR19 loci that were 626, 637, 533 and 594 bp in length, respectively. This resulted in obtaining an identical MLVA pattern for all the three strains. Compared to the *Mag* PG2 laboratory strain genome, a small difference was detected only at locus VNTR19 where the three *Mag* strains of Iran carried a 3 bp longer locus.

**Fig. 2.**
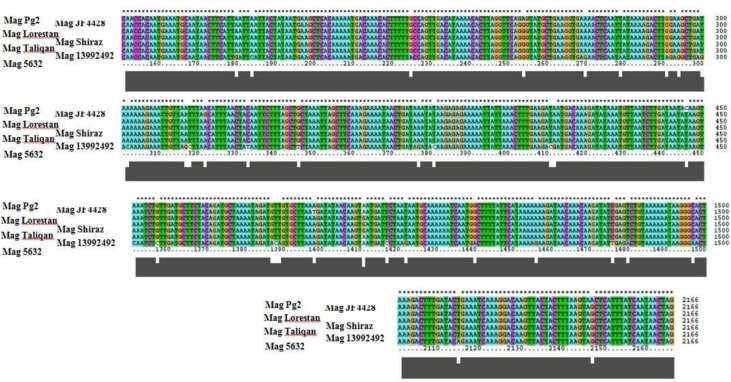
Genetic diversity at ma-mp81 gene between the three Iranian vaccine and four laboratory strains of *Mycoplasma agalctiae PG2, 5632, JF4428* and *13992492* as viewed by Clustal W. Asterisks depict identical matches of nucleotides. Numbers indicate nucleotide positions within the 2, 166 nucleotide-long ma-mp81 gene.

In analysis of the gene encoding P80 (ma-mp81), an identical substitution pattern of nucleotides between the three Iranian strains comparing to that of the *Mag* PG2 was recognized where at nucleotides 256, 446, 1411 and 2147, A, C, A and A in the genome of *Mag* PG2 substitutes with G, T, G and G in the three Iranians, respectively (Fig. 2).

## DISCUSSION

Contagious agalactiae is a poorly known disease of OIE-listed maladies where its socio-economic consequences are most felt by often poor farmers with small flocks of goats and sheep. The typically low profit margins of shepherding leads to limited interest from industry sector to fund researches aiming improve of conventional diagnosis and control measures against CA (27). This scenario happens in the Mediterranean basin and western Asia where farmers with few animals historically rear sheep and goat and continuous frequently-striking outbreaks of CA are reported. Iran along with Mongolia witness extensive burdens of CA in their farm animal populations.

The congruent observations made in this study by three approaches of VNTR, MLST and ma-mp81 gene analysis represented an identical genomic structure for the trio *Mag* strains of Lorestan, Shiraz and Taliqan.

**Fig. 3.**
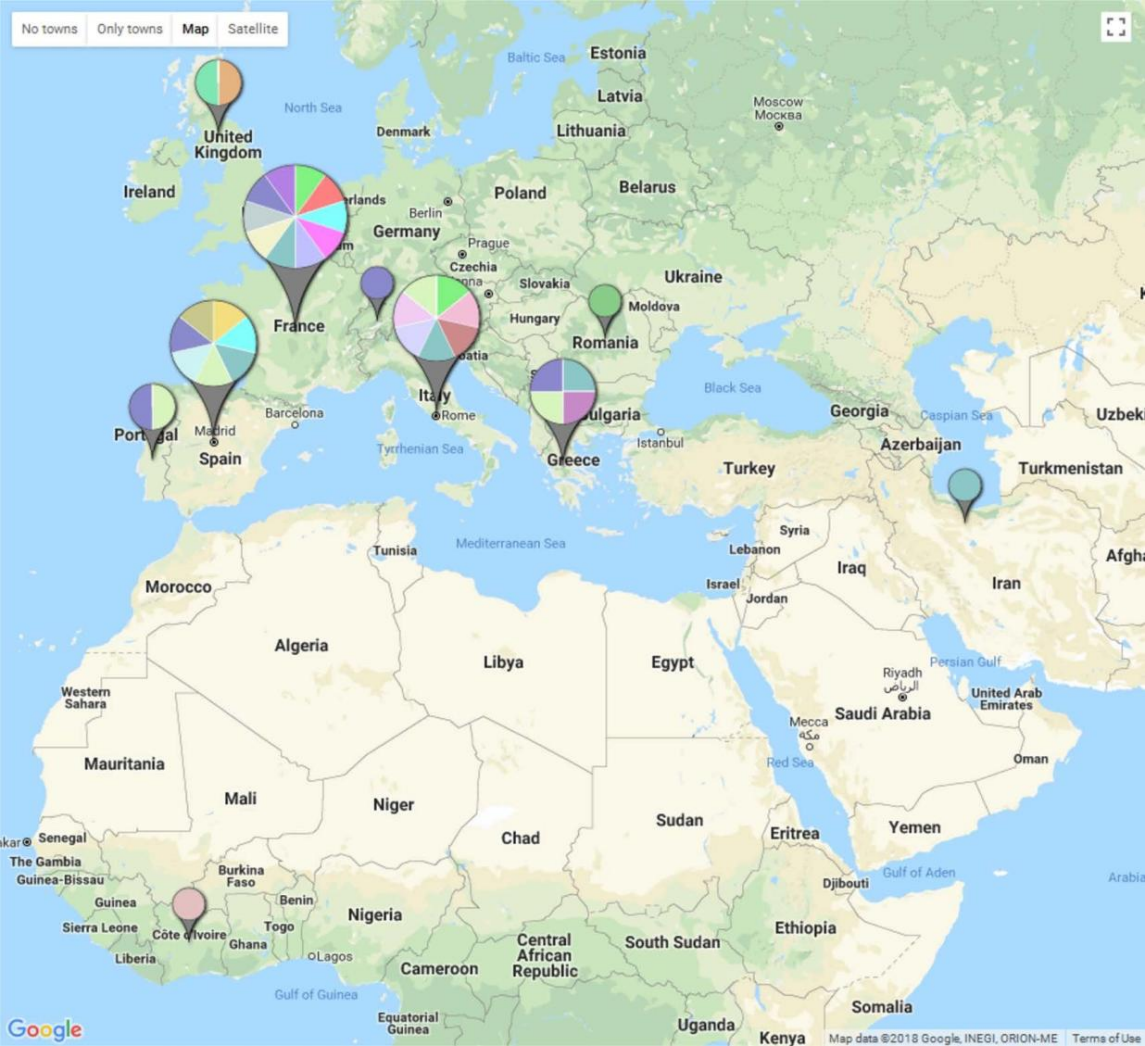
Spatial distribution of 153 world *Mycoplasma agalactiae* isolates according to their MLVA allelic patterns. Different colors of pie charts reflect different MLVA types where larger pies represent more genotyped isolates. For details see the text.

Given the long history of ruminants farming in the region and the large distance between original isolation sites of these strains, the observed identical genetic pattern of them witnessed by MLST, MLVA and ma-mp81 gene structure, is unexpected. In explanation, one can hypothesize that lack of operating efficient disease control measures, has contributed in evolution of a highly successful clone or possibly a number of closely related few clones that existed in the region for some time with a rapid rise in their frequency and eventual overwhelming of the population over the time. In southern Europe this assumption has explained the population structure of *Mag* (28). The observed similarity between the Iranian and the PG2 strain shown by MLVA and MLST approaches on the other hand, is most likely to be due to be effect of the homoplasy phenomenon or animal husbandry exercises such as animal importation (Fig. 3). Inclusion of further local isolates in next studies will help to assess these assumptions. Given the characteristics of the Iranian animal farming sector, we assume circulation of more clonal complex or complexes of *Mag* in the Iranian environment is highly expectable. A further practical approach to improve our understanding from observations made by the present study is whole genome sequencing (WGS) of the trio Iranian strains to investigate possible differences among them. Considering the outcome from such study along with possibility for circulation of new clonal complexes of *Mag* not identified yet, a good question might be whether the current vaccine needs to be re-formulated.

## References

[1] Madanat A Zendulkova D Pospíšil Z. Contagious agalactia of sheep and goats. A review. Acta Veterinaria 2001; 70: 403–412.

[2] Bergonier D Berthelot X Poumarat F. Contagious agalactia of small ruminants: Current knowledge concerning epidemiology, diagnosis and control. Rev Sci Tech 1997; 16:848–873. 9567311 10.20506/rst.16.3.1062

[3] Olaogun OM Kanci A Barber SR Tivendale KA Markham PF Marenda MS Survey of Victorian small ruminant herds for mycoplasmas associated with contagious agalactia and molecular characterisation of *Mycoplasma mycoides* subspecies *capri* isolates from one herd. Aust Vet J 2017; 95: 392–400. 28948623 10.1111/avj.12634

[4] Gomez-Martin A Amores J Paterna A De la Fe C. Contagious agalactia due to *Mycoplasma* spp. In small dairy ruminants: Epidemiology and prospects for diagnosis and control. Vet J 2013; 198: 48–56. 23759248 10.1016/j.tvjl.2013.04.015

[5] Delpy LP (1938). Infectious diseases of farm animals in Iran 1st. Razi Institute Iran.

[6] Bory G Entessar F. Etude analytique de l'immiunoprophylaxie de l'agalaxie contagieuse des chevres et des moutons. Arch Inst Razi 1959; 11: 48–52.

[7] Baharsefat M Manhouri H Yamini B. Chemical composition of *M. agalactiae*. Arch Inst Razi 1967; 19: 87–90.

[8] Mohkber Dezfouli MR Sadeghian S Javanbakht J Lakzian A. A study of occurrence and histopathology of mycoplasma infection in sheep in Tehran suburb, Iran. J Infect Dis Immun 2011; 3: 106–111.

[9] Noaman V. Identification of *Mycoplasma agalactiae* by conventional and molecular methods on small ruminants in central zone of iran. Comp Clin Pathol 2015; 24: 653–657.

[10] Heidari S Derakhshandeh A Firouzi R Ansari-Lari M Masoudian M Eraghi V. Molecular detection of *Chlamydophila abortus, Coxiella burnetii*, and *Mycoplasma agalactiae* in small ruminants' aborted fetuses in southern Iran. Trop Anim Health Prod 2018; 50: 779–785. 29260491 10.1007/s11250-017-1494-2

[11] Pooladgar A Looni R Ghaemmaghami S Pourbakhsh A Ashtari A Shirudi A. Isolation and identification of *Mycoplasma agalactiae* by culture and polymerase chain reaction (PCR) from affected sheep to contagious agalactia of Khuzestan province, Iran. Arch Inst Razi 2015; 70: 21–27.

[12] McAuliffe L Churchward CP Lawes JR Loria G Ayling RD Nicholas RA. Vntr analysis reveals unexpected genetic diversity within *Mycoplasma agalactiae*, the main causative agent of contagious agalactia. BMC Microbiol 2008; 8: 193. 18992155 10.1186/1471-2180-8-193 PMC2585094

[13] Nouvel LX Marenda MS Glew MD Sagne E Giammarinaro P Tardy F Molecular typing of *Mycoplasma agalactiae*: Tracing European-wide genetic diversity and an endemic clonal population. Comp Immunol Microbiol Infect Dis 2012; 35: 487–496. 22584004 10.1016/j.cimid.2012.04.005

[14] De la Fe C Amores J Tardy F Sagne E Nouvel LX Citti C. Unexpected genetic diversity of *Mycoplasma agalactiae* caprine isolates from an endemic geographically restricted area of Spain. BMC Vet Res 2012; 8: 146. 22920649 10.1186/1746-6148-8-146 PMC3514313

[15] Dos Santos LF Clavijo MJ Sreevatsan S Rovira A Moreira MA Pieters M. Genotyping of *Mycoplasma hyorhinis* using multiple-locus variable number tandem repeat analysis. J Microbiol Methods 2015; 111:87–92. 25661497 10.1016/j.mimet.2015.02.003

[16] Pantoja LG Pettit K Dos Santos LF Tubbs R Pieters M. *Mycoplasma hyopneumoniae* genetic variability within a swine operation. J Vet Diagn Invest 2016; 28: 175–179. 26965239 10.1177/1040638716630767

[17] Pinho L Thompson G Rosenbusch R Carvalheira J. Genotyping of *Mycoplasma bovis* isolates using multiple-locus variable-number tandem-repeat analysis. J Microbiol Methods 2012; 88: 377–385. 22261141 10.1016/j.mimet.2012.01.003

[18] Hata E Suzuki K Hanyu H Itoh M Higuchi H Kobayashi H. Molecular epidemiology of cases of *Mycoplasma californicum* infection in Japan. Appl Environ Microbiol 2014; 80: 7717–7724. 25281385 10.1128/AEM.02488-14 PMC4249231

[19] Nwankpa ND Manso-silvan L Lorenzon S Yaya A Lombin LH Thiaucourt F. Variable number tandem repeat (VNTR) analysis reveals genetic diversity within *Mycoplasma mycoides mycoides* small colony isolates from nigeria. Vet Microbiol 2010; 146: 354–355. 20580496 10.1016/j.vetmic.2010.05.020

[20] Manso-Silván L Dupuy V Lysnyansky I Ozdemir U Thiaucourt F. Phylogeny and molecular typing of *Mycoplasma agalactiae* and *Mycoplasma bovis* by multilocus sequencing. Vet Microbiol 2012; 161: 104–112. 22841405 10.1016/j.vetmic.2012.07.015

[21] Mayor D Jores J Korczak BM Kuhnert P. Multilocus sequence typing (mlst) of *Mycoplasma hyopneumoniae*: A diverse pathogen with limited clonality. Vet Microbiol 2008; 127: 63–72. 17884308 10.1016/j.vetmic.2007.08.010

[22] Harada K Kijima-Tanaka M Uchiyama M Yamamoto T Oishi K Arao M Molecular typing of Japanese field isolates and live commercial vaccine strain of *Mycoplasma synoviae* using improved pulsed-field gel electrophoresis and vlha gene sequencing. Avian Dis 2009; 53: 538–543. 20095154 10.1637/8934-052309-Reg.1

[23] Tola S Manunta D Cocco M Turrini F Rocchigiani AM Idini G Characterization of membrane surface proteins of *Mycoplasma agalactiae* during natural infection. FEMS Microbiol Lett 1997; 154: 355–362. 9311134 10.1111/j.1574-6968.1997.tb12667.x

[24] Tola S Crobeddu S Chessa G Uzzau S Idini G Ibba B Sequence, cloning, expression and characterisation of the 81-kda surface membrane protein (p80) of *Mycoplasma agalactiae*. FEMS Microbiol Lett 2001; 202: 45–50. 11506906 10.1111/j.1574-6968.2001.tb10778.x

[25] Subramaniam S Bergonier D Poumarat F Capaul S Schlatter Y Nicolet J Species identification of *Mycoplasma bovis* and *Mycoplasma agalactiae* based on the uvrC genes by PCR. Mol Cell Probes 1998; 12: 161–169. 9664578 10.1006/mcpr.1998.0160

[26] Sekhavati M Tadayon K Ghaderi R Banihashemi R Jabbari AR Shokri G “In-house” production of DNA size marker from a vaccinal *Bacillus anthracis* strain. Iran J Microbiol 2015; 7: 45–49. 26644873 PMC4670467

[27] Loria GR Nicholas RA. Contagious agalactia: The shepherd's nightmare. Vet J 2013; 198: 5–6. 23891425 10.1016/j.tvjl.2013.06.017

[28] Ariza-Miguel J Rodriguez-Lazaro D Hernandez M. Molecular characterization of *Mycoplasma agalactiae* reveals the presence of an endemic clone in Spain. J Clin Microbiol 2013; 51: 656–660. 23224102 10.1128/JCM.02835-12 PMC3553876

